# Supplementary material for: Content-rich biological network constructed by mining PubMed abstracts
Source: BMC Bioinformatics. 2004 Oct 8;5:147. doi: 10.1186/1471-2105-5-147 (PMC528731; doi:10.1186/1471-2105-5-147)
Supplement: Additional File 5 — The original Chilibot query results of the term "long-term potentiation (LTP)" and 22 other terms, limiting the latest references analyzed to the years 1990, 1995, 2000, and 2004. [file 1471-2105-5-147-S5.bz2 › chilibotAdditionalFile5/ltp2000/html/ATF.html]

 


**ATF** (Input: ATF ) 

---


**Google Searches:** Entire Web  | EDU domain only  | PDF files only

.

**External Links:** OMIM |
LocusLink
| Swissprot
| GeneCards

  
**Maps of ATF**

Simple
Complete
graph in
radiant
tree
square
layout.

**New Hypothesis !**

**Synonyms** 

- atf   [PubMed]

**Synopsis**

- These results suggest that 1 in cardiac fibroblasts activation of JNK and c Jun by Ang II is initiated by Pyk2 dependent signalings   J Biol Chem, 2000 
  [23]

- RESULTS We found a significant increase in the protein levels of Elk 1, CREB and **ATF** 2 in the cerebellar vermis of schizophrenic subjects.  Arch Gen Psychiatry, 2000 
  [23]

- These results suggest that CD28 signaling leading to the c jun promoter involves acidic sphingomyelinase,   Mol Immunol, 1999 
  [23]

- These results suggest a mechanism in which **ATF** 2 regulates insulin gene expression in pancreatic beta cells, with the transcriptional activity of **ATF** 2 being increased by an elevated concentration of calcium ions.  Diabetes, 2000 
  [20]

- The profound effect on the **ATF** family of transcription factors indicates the complexity of TGFbeta1 action on FL B malignant cells.  Exp Cell Res, 2000 
  [19]

- An effector role for methylation in Wp silencing was supported by methylation cassette assays of Wp reporter constructs and by bandshift assays, where the binding of two sets of transcription factors important for Wp activation in B cells, BSAP Pax5 and CREB **ATF** proteins, was shown to be blocked by methylation of their binding sites.  J Virol, 2000 
  [17]

- The analysis of two distinct mitogen activated protein kinase pathways shows that stress activated protein kinase Jun N terminal kinase activation, resulting in the phosphorylation of **ATF** 2, c Jun, and JunD, is required NOT only for the IL 1 but for the TPA dependent induction, while the extracellular signal related kinase 1 ERK 1 and ERK 2 activation is involved in the TPA  Mol Cell Biol, 1999 
  [17]

- CREB2 called ATF4, TAXREB67, or C **ATF** is an evolutionarily conserved member of the CREB **ATF** family of basic leucine zipper transcription factors.  Dev Biol, 2000 
  [17]

- **ATF** 2 at Ser 69 71, forming a complex with Myc for DNA binding at CRE motif.  Shi Yan Sheng Wu Xue Bao, 1997 
  [13]

- This region binds RARs and RXRs nuclear factors, CREB **ATF** factors, and PPARgamma despite the absence of a consensus peroxisome proliferator response element.  J Biol Chem, 2000 
  [12]

- This element is recognized by members of CREB **ATF** as well as C EBP family of transcription factors.  Mol Cell Biochem, 2000 
  [12]

- Together, these findings implicate CREB **ATF** proteins in essential functions during molting and metamorphosis.  Genetics, 2000 
  [12]

- Subsequent activation of the cAMP PK A signaling pathway results in enhanced synthesis and binding of the transcription factor CREB 1 **ATF** 1 to the cAMP responsive elements within the IE enhancer.  Virology, 2000 
  [11]

- Fifth, Mas and Rac1 stimulated transcription from common DNA promoter elements NF kappaB, serum response factor SRF, Jun **ATF** 2, and the cyclin D1 promoter.  Mol Cell Biol, 1998 
  [10]

- RESULTS Viruses previously designated group D that contain two mutations in the activating transcription factor cAMP response element **ATF** CRE in the LMP 1 promoter, which are known to decrease promoter activity greatly, were significantly less frequent in Hodgkin s disease than in both infectious mononucleosis p = 0.0081 and asymptomatic EBV carriers p = 0.0084 .  Mol Pathol, 2000 
  [10]
